# Chelation: Harnessing and Enhancing Heavy Metal Detoxification—A Review

**DOI:** 10.1155/2013/219840

**Published:** 2013-04-18

**Authors:** Margaret E. Sears

**Affiliations:** Children's Hospital of Eastern Ontario Research Institute, 401 Smyth Road, Ottawa, ON, Canada K1H 8L1

## Abstract

Toxic metals such as arsenic, cadmium, lead, and mercury are ubiquitous, have no beneficial role in human homeostasis, and contribute to noncommunicable chronic diseases. While novel drug targets for chronic disease are eagerly sought, potentially helpful agents that aid in detoxification of toxic elements, chelators, have largely been restricted to overt acute poisoning. Chelation, that is multiple coordination bonds between organic molecules and metals, is very common in the body and at the heart of enzymes with a metal cofactor such as copper or zinc. Peptides glutathione and metallothionein chelate both essential and toxic elements as they are sequestered, transported, and excreted. Enhancing natural chelation detoxification pathways, as well as use of pharmaceutical chelators against heavy metals are reviewed. Historical adverse outcomes with chelators, lessons learned in the art of using them, and successes using chelation to ameliorate renal, cardiovascular, and neurological conditions highlight the need for renewed attention to simple, safe, inexpensive interventions that offer potential to stem the tide of debilitating, expensive chronic disease.

## 1. Introduction

The living body is full of chelates; metals bound with two or more coordination bonds. Metals of oxidation state greater than one (i.e., a charge of +2 or more) are predominantly bound in tissues by ionic (in skeletal minerals) or coordination bonds (e.g., bound to albumin, enzymes, small peptides, and amino acids such as cysteine, methionine, and selenomethionine). This was extensively reviewed by Apostoli et al. [1]. 

Cadmium [2–5], lead [6–8], and mercury [9–12] have no essential biochemical roles, but exert diverse, severe toxicities in multiple organ systems as they bind in tissues, create oxidative stress, affect endocrine function, block aquaporins, and interfere with functions of essential cations such as magnesium and zinc. Toxic metals pose particular risks to the very young, as exposures early in life compromise development, with lifelong physical, intellectual, and behavioural impairments. In adults, major chronic diseases [13], including cardiovascular and renal disease, and neurological decline, are also strongly associated with toxic elements. The International Agency for Research on Cancer (IARC) classifies cadmium as a known carcinogen, inorganic lead a probable carcinogen, and methylmercury a possible carcinogen [14].

As research progresses, harms more subtle than acute poisoning are seen at lower and lower body burdens of heavy metals. For example, early lead exposure is now found to cause IQ decrements at a blood level below 2 *µ*g/dL [15]. The blood lead reference value at which the US Centers for Disease Control action recommends investigation and remediation of a child's environmental exposures is 5 *µ*g/dL, while chelation is recommended at nine times that level above 45 *µ*g/dL [16]. 

Modern mercury and cadmium exposures are frequently via oral routes, prompting advisories regarding fish (e.g., U.S. Environmental Protection Agency [17]), seafood and wildlife consumption (e.g., Canadian Aboriginal Affairs [18]), as well as cigarette smoke (also noted by Aboriginal Affairs; cadmium is but one toxic component). Lead may also originate in old drinking water supply pipes.

Toxic metals are ubiquitous in our environment, and thus in ourselves, at higher than historical levels. Exposures [5, 8, 12, 19] include the activities and legacies of mining and toxic wastes, lead in paint and gasoline, ongoing emissions from industrial and electricity-generating (particularly coal-burning) activities, chemicals in everyday products, and novel technologies such as nanomaterials containing toxic elements like cadmium [2].

Biological mobility, tissue concentrations, and excretion of metals are determined by oxidation state, solubility, a complex set of equilibria between complexing sites, as well as active transport through membranes [1]. Chelation is central to natural detoxification of heavy metals, via formation of complexes, particularly with glutathione and other small molecules, and their excretion [20]. 

This manuscript stems from a large scoping review regarding arsenic, cadmium, lead, and mercury, funded by the Canadian Institutes of Health Research. Multiple online literature searches included a comprehensive list of terms for the toxic elements and peer-reviewed search strategies, to search research publication databases, as well as governmental (e.g., Environment Canada, US Environmental Protection Agency) and nongovernmental (e.g., World Health Organization) sources, described previously [21]. Expert opinion was solicited via email, during a conference call, and during a two-day conference in Toronto (February 2011). Clinical toxicologists at Canadian Poison Control Centres were surveyed to gather information about screening, experiences, and preferred chelators for each toxic element. Ethics approval was obtained from the Children's Hospital of Eastern Ontario Research Institute, Ottawa, Canada.

In this paper, measures to support natural detoxification pathways involving chelation, as well as use of pharmaceutical chelators are examined. Historical adverse outcomes, lessons learned in the art of using chelators, and successes using chelation to ameliorate renal decline, cardiovascular disease, and autism in children are reviewed.

## 2. Chelation Background

“Chelation,” from “chelos” the Greek word for claw, involves the incorporation of a mineral ion or cation into a complex ring structure by an organic molecule, the chelating agent. Typically, electron-donor atoms on the chelating molecule include sulphur, nitrogen, and/or oxygen. 

The strength of the chemical bonds within coordination complexes that are formed between chelators and metal ions depends upon the elements involved and details of the stereochemistry. With a variety of metal ions that could bind competitively with the chelator (e.g., calcium, magnesium, zinc, copper, manganese, and other metals, that typically exceed concentrations of toxic elements), the identity of the metal predominately bound by a chelating agent depends both upon accessibility of the chelator to the tissues, how strongly the metal is already bound in the tissues, how strongly the metal binds to the chelator, and to some extent the relative quantities of various ions [1]. Chelators have the effect of mobilizing metals from tissues and maintaining the chelate moiety during circulation to the kidneys for excretion in the urine, and to the liver for excretion in the bile. There are significant concerns related to enterohepatic recirculation and reabsorption in the kidney [22].

Another consideration is solubility of the chelate, in water and in lipids. Aqueous solubility facilitates transport within the blood and excretion via the kidney, while a lipophilic chelator may exhibit greater penetration of cellular membranes (including those within the central nervous system) to chelate intracellular elements. A lipophilic chelator may also be excreted in greater quantities via the bile. These generalities may be modified by active transport of intracellular metal complexes via “drug resistance proteins” [23–26].

## 3. Roles of Chelation in Natural Toxicokinetics

Metal binding proteins, including metallothioneins, are potent chelators for heavy metals and are central to the natural response of the body to these toxic elements [27, 28]. Glutathione is another potent chelator involved in cellular response, transport, and excretion of metal cations and is a biomarker for toxic metal overload [29–31].

Not only animals, but also plants produce chelating compounds [32], and metallothionein content of foods may affect bioavailability as well as metabolism of toxic metals such as cadmium [33]. 

Some foods have been suggested to reduce absorption or reabsorption of toxic metals and to support natural detoxification pathways. 

(i) Dietary fibres from various food products, including bran from grains as well as fruit, have been evaluated as an alternative or adjunct to chelation therapy with the aim to interrupt enterohepatic recirculation [34–36] and to modulate intestinal flora [37], with findings of reduced levels of mercury in the brain and blood. Caution is merited regarding soluble fibre; in contrast to protection offered by insoluble fibre, flax seed resulted in increased intestinal absorption of cadmium [38]. 

(ii) Other natural polymers have also been gaining attention as potential adsorbents of heavy metals, such as algal polysaccharides alginate [39] and chlorella [40]. Modified citrus pectin plus alginate products have been used successfully to reduce lead and mercury in case studies [39]. Poly(*γ*-glutamic acid), an edible and biodegradable biopolymer, has been produced extracellularly during fermentation of *Bacillus* species; its *α*-carboxyl groups conjugate a variety of compounds including metal cations [41]. 

(iii) Given that toxic metals have great affinity for sulphur-containing peptides, diets rich in sulphur-containing foods such as alliums (e.g. garlic [42]) and brassicas (e.g., broccoli [43]) have been suggested for effects on glutathione, with hopes for symptomatic improvement and enhanced excretion. Garlic prevented cadmium-induced kidney damage [44] and decreased the oxidative damage due to lead in rats [45].

(iv) Cilantro (leaves of *Coriandrum sativum*), a popular culinary and medicinal herb, gained attention when a soup was reported to enhance mercury excretion following dental amalgam removal and remains popular despite limited evidence [46]. In animals, it decreased lead absorption into bone and inhibition of the delta-aminolevulinic acid dehydratase (ALAD) enzyme [47]. Less encouragingly, in a recent trial in 3- to 7-year old children exposed to lead, a cilantro extract was as effective as placebo in increasing renal excretion (improvements across treatment and placebo groups were ascribed to improved diet during the intervention) [48].

Several supplements are also in use to address metal toxicities. 

(i) Taurine [49–51] and methionine [52] are sulphur-containing amino acids. They are rich in membranes particularly of excitable tissues, and they decrease oxidative stress markers resulting from heavy metal exposure. Practitioners also report using taurine for 6 weeks or so prior to hair analyses, to boost levels and improve detection.

(ii) Alpha lipoic acid is a powerful antioxidant that regenerates other antioxidants (e.g., vitamins E and C, and reduced glutathione) and has metal-chelating activity. Both fat and water soluble, it is readily absorbed from the gut and crosses cellular and blood-brain membrane barriers [22, 53]. Clinical experience is that it must be used carefully as it poses particular risks of redistribution of metals.

(iii) N-acetyl-cysteine (NAC), an orally available precursor of cysteine, is a chelator of toxic elements and may stimulate glutathione synthesis, particularly in the presence of vitamins C and E [54–56].

(iv) Glutathione is not recommended to be administered orally as it undergoes digestion; however novel modes of delivery such as liposomal and prodrug preparations are emerging [57]. It may be administered intravenously, in creams and via nebulizer. Glutathione is an important physiological chelator, and the reduced form of glutathione protects cells from reactive oxygen species associated with heavy metals [58–61].

(v) Selenium is an important essential element, that is present at a broad range of levels across populations. The selenide ion forms an extremely stable, insoluble compound with mercury, and provides relief of mercurialism symptoms. On the face of it, selenide might not be compatible with chelation, as the two agents may counter the effectiveness of one another [62]; however, selenium may be incorporated in organic molecules, and organic selenium/mercury complexes may be transported through membranes. Selenium depletion in the face of mercury exposures also depletes seleno-enzymes. In humans, organic selenium supplementation was beneficial in a controlled trial among 103 mercury-exposed villagers [63]. A selenium yeast product increased mercury excretion and decreased oxidative stress-related biomarkers urinary malondialdehyde and 8-hydroxy-2-deoxyguanosine [63].

Overall, a number of studies have investigated the effects of micronutrients such as vitamins, sulphur-containing amino acids, antioxidants, and essential minerals on kinetics and adverse effects of toxic elements [64–68]. Nutritional status affects uptake, as toxic cations are transported by proteins for essential nutrients such as magnesium, zinc, and iron, putting those who are malnourished at greater risks for toxicity [2, 33]. This suggests potential for dietary preventive public health interventions. For example, in animals calcium deprivation enhanced absorption of lead and cadmium [69], while magnesium and zinc supplementation blunted absorption of cadmium [2]. Calcium supplementation reduced lead mobilization from maternal bones during pregnancy and lactation, protecting the newborn and infant [70–72]. In children, iron supplementation blunted lead accumulation [73]; however, mineral supplementation and school meal programs should not divert attention from the paramount importance of removal of the sources of exposure [74–76]. 

## 4. Pharmaceutical Chelators

Pharmaceuticals that chelate metal ions in solution are small organic molecules that typically form coordination complexes involving sulphur, oxygen, and/or nitrogen atoms. 

Drug information from the US National Library of Medicine for five chelating agents used most commonly for the treatment of humans intoxicated with heavy metals and metalloids is summarized below, and in Table 1 [56].

Dimercaprol (British Anti-Lewisite, BAL), the first antidote to an arsenical nerve gas, is a dithiol prepared in an oil base and given only by intramuscular injection (painful). It has a narrow therapeutic window and is commonly prepared with peanut oil, posing a risk of allergic reaction. 

BAL has been largely supplanted by dimercaptosuccinic acid (DMSA or succimer) and dimercaptopropane sulfonate (DMPS), that were extensively researched in Russia, China, and Japan, a half century ago [77]. These dithiols, with greater water solubility, are being administered as oral, intravenous, suppository, or transdermal preparations. The absorbed dose is excreted with a half-life of approximately 3 hours; longer in children and people with mercury toxicity.

Oral administration of DMSA may be limited by intestinal dysbiosis. Oral absorption is approximately 20%, with most DMSA in plasma being protein bound (95%, mainly to albumin); only a very small amount is present as free drug. DMSA is extensively metabolized in humans to mixed disulfides of cysteine. Ten to 25% of an orally administered dose of DMSA is excreted in urine; the majority within 24 hours and most as DMSA-cysteine disulfide conjugates. The remainder is largely eliminated in the faeces [77–80]. DMSA increases urinary excretion of arsenic, cadmium, lead, methylmercury, and inorganic mercury, with removal from animals' brains of lead and methylmercury. Successful dialysis of methylmercury-DMSA complexes has been reported. Excretion of essential metals like zinc, iron, calcium, and magnesium is much less than with CaNa_2_EDTA, with potentially higher losses of copper in humans. Although frequently administered orally, intravenous, rectal, and transdermal routes are in clinical use. A rare side effect is mucocutaneous eruptions and toxic epidermal necrosis, that resolves when the medication is stopped.

DMPS oral absorption is approximately 39%, higher than that of DMSA [81]. Solutions are relatively stable, so DMPS is administered intravenously more frequently than DMSA. DMPS is rapidly converted to a disulphide form and is excreted largely in the urine as acyclic and cyclic disulfide chelates, with an overall half-life of approximately 20 hours following intravenous administration [81]. A significant proportion is also excreted in bile. DMPS increases urinary excretion of arsenic, cadmium, lead, methylmercury, and inorganic mercury. In a study of the DMPS challenge test there was significantly increased excretion of copper, selenium, zinc, and magnesium, necessitating replenishment of these essential minerals orally or intravenously before and after treatment [82]. 

In comparing the efficacy of the dithiol chelators in animals, DMSA was superior in removal of methylmercury, including from animal brains. Although DMPS did not affect levels in the brain, it was superior at removing methylmercury from the kidney [77]. In mice, cadmium was removed more effectively by DMSA than DMPS [83].

CaNa_2_EDTA is not metabolized and EDTA chelates are rapidly excreted, principally in the urine. With only oxygen atoms for coordination bonds, EDTA binds lead and cadmium strongly, eliminating them in the urine. Clinical experience is that CaNa_2_EDTA will result in increasing mercury excretion once other more well bound minerals such as lead and cadmium are depleted. Overall CaNa_2_EDTA causes greater losses of essential minerals than DMSA or DMPS.

Penicillamine binds with copper and is used for Wilson's disease. It will mobilize arsenic, cadmium, lead, and mercury, but it is generally not a drug of choice. It was inferior to DMSA and DMPS in removal of methylmercury from animals, with no effect on levels in the brain [77].

Canadian clinical toxicologist questionnaire respondents indicated that their preferences for chelation therapy for chronic toxicity would be DMPS or DMSA for arsenic; EDTA plus BAL, or as a second line medication penicillamine for cadmium; DMSA orally (or possibly EDTA plus BAL for acute exposure) for lead; and DMSA or DMPS (or possibly BAL for acute exposure) for mercury. 

### 4.1. Roots of Chelation Controversies

#### 4.1.1. EDTA Concerns

Three deaths associated with chelation therapy have been reported, related to hypocalcemia resulting in cardiac arrest after use of Na_2_EDTA [84]. These were in fact drug errors and should not reflect on the safety of CaNa_2_EDTA, the form generally indicated for chelation of toxic metals [85]. 

CaNa_2_EDTA is distributed mainly in the extracellular fluids and one of its major perceived drawbacks is that of redistributing lead from other tissues to the brain. In one study, treatment with DMSA after exposure to inorganic mercury caused an elevation of mercury in motor axons, likely due to redistribution of mercury, which was mobilized from nonneural tissues such as the kidneys and liver [86]. Mixed reports indicate that EDTA does not cross the blood-brain barrier, but this is in contrast to reports that EDTA may cause increased symptoms of lead poisoning or mercurialism [87]. 

Transient increases in hepatic transaminase activity have been reported with CaNa_2_EDTA, DMSA, and DMPS, but hepatic toxicity resolves with discontinuation of the medication. Skin lesions associated with CaNa_2_EDTA may relate to zinc deficiency. 

#### 4.1.2. Chelation Therapy in Children

A single trial published in the New England Journal of Medicine (2001) is cited by authorities who recommend that chelation therapy be used only at highly elevated blood lead levels in children [88]. In an early, ambitious trial using DMSA chelation therapy in 780 children enrolled in the “Cincinnati cohort,” blood lead levels were temporarily lowered in children receiving the medication compared with the control group; however, at 36-month followup blood lead levels in the treated children had rebounded. At this 3-year mark, there were no significant differences between treatment and control groups in terms of blood lead levels nor neurocognitive outcomes [89]. This trial used a very aggressive protocol, with 26 days of therapy for one, two, or three rounds. Currently, chelating agents are typically administered for multiple shorter periods, with time between courses for the body's minerals to become repleted. This aggressive therapy could very well have depleted essential minerals from this vulnerable population (poor, inner-city, and black/Hispanic children). The vitamin and mineral supplementation may have been inadequate and may have been countered by concomitant administration of the chelator (doses and adherence to treatment for supplemental minerals were not reported). Shannon et al. have offered similar criticisms [90]. 

A trial of chelation therapy to treat autism registered on the US National Institutes of Health (NIH) http://www.clinicaltrials.gov website is indicated as “completed,” with the last update October 13, 2009 [91]. When contacted for an update, the NIH representative replied that the trial had been cancelled before recruitment, because some adverse effects were observed in a study of 120 rats [92]. This study by Stangle et al. clearly demonstrated that a single three-week course of high dose DMSA treatment ameliorated learning, attention, and arousal regulation in rats exposed to lead during a period from early postpartum to late adolescence. The treatment also reduced lead levels in both the blood and brain. What prompted cancellation of the autism trial was detection of a potential adverse drug effect in the form of adverse cognitive effects among *unexposed* rats that were treated with DMSA, compared with unexposed, untreated rats.

This pivotal animal study led to cancellation of a large, much-publicized trial in children. In assessing the relevance for the trial cancellation and to clinical practice, several issues are pertinent. Adverse effects of drugs are common, which is why drugs are not usually given without an indication that they are needed. (Pre- and postchelation challenge testing to assess excretion of both toxic and essential elements is discussed below). In the Stangle et al. study no mineral supplementation was provided, and no minerals other than lead were analysed. DMSA is well known to enhance excretion of many elements, notably zinc [93]. Zinc deficiency impairs neurocognitive development in the young [94, 95]. In addition, Stangle et al.'s rats were treated using an “aggressive” protocol, with 50 mg/kg/day DMSA for 21 days; a dose that is much higher than the US Food and Drug approved maximum label dose of 30 mg/kg/day [93], that is typically used for less than a week at a time in children [96]. It is probable that detrimental effects attributed to DMSA resulted from deficiency of essential elements, an effect that is eminently avoidable.

In summary, the Stangle et al. study violated important current clinical practices by administering the drug at a high dose, over an extended period of time, when there was no indication of need; and failing to assess essential minerals loss and ensuring that minerals were appropriately supplemented to avoid health consequences.

### 4.2. Chelation in Various Tissues and Redistribution

Chelating agents are fairly rapidly excreted over a few hours or days. In contrast, toxic elements may have accumulated over long periods of time and partitioned into various bodily compartments, not all being equally accessible to chelating agents. Commonly a chelating agent will mobilize the most readily available metals first, typically in the plasma, kidney, liver and then to a lesser extent bone and central nervous system. As discussed above, toxic metals in the nervous system are best addressed conservatively, with repeated, modest treatments and the use of multiple agents. With repeated doses the most readily accessed “pools” of toxic elements will be depleted, but reequilibration slowly replenishes the toxic elements in more accessible body compartments. This is evident in the rebound of levels in the blood, following discontinuation of a chelator, which highlights two important facts.

(i) Blood and urine are poor surrogates to measure the toxins accrued over the lifetime (body burden). The common laboratory measures of urine, blood, and hair indicate exposures in recent days or months, and to a lesser extent kidney burden. 

(ii) Toxic elements sequestered in bone and soft tissues are not completely immobilized; they migrate back to the bloodstream and hence to tissues where they will again exert toxic effects. It is important to gain a greater understanding of the quantities of biologically accessible toxic elements within the body that are not necessarily reflected in baseline blood or urine levels, before chelation provocation.

(iii) Introduction of a chelating agent into the body causes shifts of both essential and toxic cations. Increased symptoms commonly reported with aggressive initiation of chelation therapies are cited as a contraindication to any use of chelators. Improvements are nevertheless reported with low initial doses and gradual titration according to patient tolerance (characterized as a marathon rather than a sprint).

### 4.3. Testing to Identify Toxic Metals and to Follow Progress of Therapy

Toxicologically significant levels of toxic elements may not be predictable from exposure history, as relevant exposures may not be queried, recognized, or remembered. Furthermore, mobilization of metals from various compartments in the body could occur due to certain stressors such as disease, trauma, starvation, pregnancy, time of life (e.g., menopause), and extreme emotional impacts. Depending on a person's constitution, genetic make-up, diet, lifestyle, and sensitivities, a patient could be suffering from toxic metal effects without having a clear history of exposure. It is a common clinical experience that chronic conditions (e.g., neurological disturbances in a teacher who ate considerable quantities of tuna [97]) are linked to the causative toxic elements only following a test identifying elevated levels.

It is difficult to draw conclusions about adverse health effects of metals without assessing net retention, that is, the differences between the rates of assimilation and excretion of metals over the lifetime. In addition, clinicians require information to guide therapy. Most commonly, metals are analysed in urine, whole blood, red blood cells; less commonly hair; or rarely toenails.

One of the most effective methods to evaluate net retention, or at least the biologically readily available metal load, is to compare the levels of metals in urine before and after the administration of a pharmaceutical chelating agent such as CaNa_2_EDTA, DMSA, or DMPS [98]. Variously known as “mobilization,” “chelation challenge,” or a “provocation” test, this procedure is not universally accepted as standard of care. Criticisms have included risks of the chelating drugs, and inappropriate comparisons of the provocation results with population norms rather than with patient baseline concentrations [85]. Indeed, some go so far as to say that any testing for metals when the exposure has not been identified; that is, when there is no reason for suspicion based upon known environmental history that toxins may be elevated, is inappropriate because of the possibility that false positives may lead to inappropriate, ineffective therapies and their attendant risks [99]. The use of chelation for diagnostic purposes, following dental amalgam removal or in asymptomatic patients with baseline urine or blood levels approximating population norms was deemed inappropriate in 2005 by staff of the Agency for Toxic Substances and Disease Registry [85]. Another criticism of use of a provocation test to judge net retention is the lack of a standard protocol, and laboratory reference ranges or guidance for interpretation of results [100]. Nevertheless, these shortcomings do not fundamentally invalidate the concept; work in this regard has started. Hansen et al. established such norms for protocol involving an oral DMPS test with four hour urine collection, among 2223 citizens in Luxembourg [101].

Pre- and postchallenge testing may allow the clinician to identify which chelating agent is the most effective for the patient, and if oral agents are employed, possible absorption or tolerance problems may be identified. An open research question has to do with changes in metals excreted over an extended course of chelation treatments; whether in a person with high levels of multiple metals, one will be preferentially chelated initially, with a second then third being excreted over time with repeated treatments. This research would aid interpretation of chelation challenge tests, as well as enhance knowledge of chelation therapy itself.

Comparison of baseline and provoked urine levels is entering standard practice and was used to determine inclusion in a trial of chelation therapy for children with autism [96]. In this trial, however, a few children experienced worsening symptoms. Such worsening is ascribed to redistribution of toxic metals, with insufficient excretory mechanisms in place, leading some practitioners to prefer unprovoked analyses up front, in sensitive, fragile patients. Therapy may be guided by parental, caregiver, and patient observations.

### 4.4. Therapeutic Benefits

Chelation therapy is established as an effective treatment for acute and higher exposure poisoning, according to the drug labels. Examples of reports using chelation agents for high occupational or environmental exposures include the following.

- DMSA chelation therapy increased lead excretion on average by a factor of 12 and rapidly reversed lead related symptoms (largely neurological and gastrointestinal) in a case series of 17 lead-poisoned adults [102]; these authors also reviewed effectiveness of DMSA.
- The same group reported a case of a jeweller with extensive neurological symptoms of mercury poisoning, reversed with DMPS treatment [103].
- A trial of oral DMPS therapy in the Philippines provided two weeks of treatment in a community highly exposed to mercury used for artisanal gold mining [104]. Most participants experienced multiple significant neurological improvements. This trial was remarkable for the extensive testing conducted in this remote location, as well as near-perfect compliance, as the midwife distributed the medication. This report is high quality, with careful descriptions of the intervention, inclusion, dropouts, and results.

The effective use of chelation in patients with lower levels of accumulation of toxic elements is not as widely recognized, but positive trials are being reported. 

(i) In a randomized, double-blind controlled trial conducted by Adams et al., reductions in measures of the severity of autism were associated with the difference in urinary excretion of toxic metals before and following treatment with DMSA, demonstrating both a significant positive association between the severity of autism and the body burden of toxic metals, and efficacy of reduction of this body burden in improving symptoms [96]. An inclusion criterion for the trial was elevated body burden of one or more toxic elements, determined using chelation challenge testing. The initial three days of treatment for this inclusion screening was sufficient to improve glutathione and platelet levels in children with autism [105].

(ii) A concern with chelation therapy is that renal insufficiency may be a contraindication for therapy. The opposite appears to be the case. In a randomized, controlled study of 64 patients with chronic renal insufficiency with elevated body burden of lead and without diabetes, three months of CaNa_2_EDTA weekly infusions resulted in slowing or reversing degeneration in the chelation group. Following 24 further months of treatment in 32 patients with elevated body lead burdens, glomerular filtration rate improved among the treatment group and decreased in controls. The rate of decline among those not treated during followup was lower among previously treated patients. Cost of therapy was approximately $3750 per patient, compared with a cost of $61,000 for hemodialysis over a similar time frame for end stage renal failure [106]. In a smaller trial in patients with type II diabetes, body lead burden was a strong predictor of rate of renal function decline. Chelation therapy halved decline during three months of treatment but kidney function worsened in both groups during nine months further followup without treatment [107]. Of note, no other toxic elements were measured during this research, so it is unknown to what extent other nephrotoxins such as cadmium or mercury may have also played roles.

(iii) A 1955 report that patients with ischemic heart disease had improvement in angina and other cardiovascular symptoms while undergoing EDTA chelation therapy for lead poisoning sparked long, ongoing interest in the prevention and treatment of cardiovascular disease [108]. EDTA chelation therapy treatment for atherosclerosis has been a controversial subject of debate. While early anecdotal evidence suggested significant clinical symptomatic improvements, the five clinical trials identified in a recent meta-analysis used small populations with different clinical syndromes, measured different outcomes, and yielded no overall evidence of benefit [109]. 

The Trial to Assess Chelation Therapy (TACT) [110] was a US National Institutes of Health sponsored, randomized, double blind, placebo-controlled clinical trial, evaluating the benefits and harms of EDTA chelation therapy in 1708 nonsmokers aged 50 and older who had an acute myocardial infarction more than 6 weeks prior to enrolment and were otherwise medically stable. The protocol was recommended by the American College for Advancement in Medicine, the largest physicians' organization in America practicing chelation. Treatment included 40 3-hour infusions of a multicomponent Na_2_EDTA solution, plus an oral, high-dose multivitamin/mineral supplement on nonchelation days. The primary endpoint was a composite of all-cause mortality, myocardial infarction, stroke, coronary revascularization, and hospitalization for angina [111]. The success of this trial was reported at the American Heart Association meeting in November 2012. Three years after treatment, the risk of the combined endpoint was reduced by 18% in the group receiving EDTA (*P* = 0.03) compared with placebo. Among participants with diabetes and those who had experienced anterior myocardial infarctions, the combined endpoint was reduced by 39% (*P* = 0.002). Of equal importance, there was no difference between groups in serious adverse events. Hypocalcemia and transient renal function impairment, the two complications that had been reported in early studies using primitive protocols, did not occur at all. TACT proceeded despite detailed criticisms [112], but unfortunately excretion of toxic elements was not assessed during this trial. Thus, participants in whom chelation would potentially have been indicated on the basis of higher body burdens of toxic elements known to be associated with cardiovascular disease were not identified, and it is unknown if additional benefit may have accrued with additional treatment, among those with remaining significant body burdens of heavy metals.

### 4.5. Other Potential Pharmaceutical Chelators

Monoisoamyl DMSA (MiADMSA) is a potential drug candidate under development. In young rats exposed to lead or arsenic, MiADMSA was found to potentiate the synthesis of metallothionine in liver and kidneys and glutathione in liver and brain, along with significantly reducing the glutathione disulfide levels in tissues. MiADMSA is capable of mobilizing intracellularly bound cadmium and is seen to provide an indirect antioxidant effect by removing cadmium from the site of deleterious oxidation reactions [86].

Analogues of DMSA are capable of crossing biomembranes and are more effective in reducing arsenic burden in acute and subchronic intoxication. Monoesters may be preferred over DMSA diesters owing to their higher efficacy against arsenic intoxication and lower toxicity of the drug [86].

N-(alpha-L-Arabinofuranos-1-yl)-L-cysteine, stereoselectively prepared from L-arabinose and L-cysteine, is an experimental chelator which has been shown to have good intra- and extracellular mobility as well as little effect on the level of essential minerals when used in mice [113].

Older drugs known as “metal protein attenuating compounds” (MPACs) such as clioquinol are weaker chelators, thought to modulate copper and zinc in the brain, removing it from plaque and tangles. As with the TACT trial focusing on calcium, the focus has been on known physiologically essential metals, and little thought and research has been devoted to possible effects of MPACs on toxic metals. The hypothesis that MPACs may be acting on toxic as well as essential metals merits further investigation, as clioquinol and vitamin B were found to reduce lead accumulation and to rescue brain plasticity in rats [114].

### 4.6. Combination Therapies

Combination therapy is an approach to enhance metal mobilization from the body, reduce individual doses of chelators, and lessen redistribution of toxic metals from one site (e.g., bone or liver) to more sensitive sites such as the brain (discussed above). There are a large number of possible agents, which are being tested in animal research. This is an area ripe for research; here are a few examples.

Animals chronically exposed to lead experience redistribution from bone to soft tissues including the brain following CaNa_2_EDTA. This is also seen in humans, leading to the recommendation that EDTA chelation be followed by a short course of DMSA [115]. Indeed, the recommendation to combine EDTA with thiol chelators was reported decades ago [116]. In lead-treated rats, a DMSA and CaNa_2_EDTA combination was superior to either drug on its own, or to DMPS alone or in combination with CaNa_2_EDTA, in depleting organ and bone lead, normalizing lead-sensitive biochemical measures with no redistribution of lead to any other organ. DMSA was the only drug that resulted in decreased brain lead levels [117].

Coadministration of DMSA and monoisoamyl DMSA (MiADMSA) at lower doses was most effective not only in reducing arsenic-induced oxidative stress but also in depleting arsenic from blood and soft tissues compared to other treatments [86].

Supplementation with antioxidants and small molecules containing thiol groups, along with chelating agents may be beneficial in increasing toxic metal mobilization and excretion, with improvement of biochemical variables [118]. For example the following.

- Taurine, when coadministered with DMSA or MiADMSA, helped to further reduce total body burden of arsenic and lead [119].
- NAC forms coordination bonds between metals and its thiol group. The thiol may also reduce free radicals. Combined administration of NAC and DMSA after arsenic exposure led to a significant reduction of oxidative stress biomarkers, as well as to removal of arsenic from organs [120].
- The research group led by Flora has investigated toxic metals extensively in animals, and reviewed combinations of antioxidants and other agents in addition to chelators, including vitamins, NAC, taurine, lipoic acid [20], and liposomal glutathione [60, 61].

### 4.7. Clinical Approaches

Management of patients in whom low dose chronic toxic metal exposures are contributing to chronic illnesses presents a significant challenge to the health care provider. Irritable bowel syndrome, fatigue, autism spectrum disorders, cognitive impairment, allergies, environmental sensitivities, or soft neurological signs like tremor, imbalance or depression may be multifactorial in origin. Such patients' clinical situations are unique and complex, necessitating multiple therapeutic strategies. Individualized therapy is provided according to the best available evidence, clinical judgment, and patient preference, in order to maximize benefit and minimize risk [19]. A thorough work-up is used to identify underlying factors, such as allergens and gluten intolerance, that are addressed by avoidance, food intolerance identification and remediation (rotation and elimination diets) and pre- and probiotics for intestinal dysbiosis. As a result of intestinal malabsorption patients may present with nutritional deficiencies, which can be addressed through dietary counselling, oral supplementation with vitamins and minerals, and intravenous supplementation using a mixture such as Myers Cocktail, to which glutathione may be added. It should be noted that there is concern about endocrine disrupting di (2-ethylhexyl) phthalate (DEHP) leaching from vinyl intravenous bags and tubing [121].

An extensive environmental exposure history is used to identify xenobiotic exposures [122], so that sources may be recognized (e.g., occupational exposures), remediated (e.g., dust from lead-based paint) and avoided (e.g., consumption of high-mercury fish, or smoking). Once the sources of toxins are removed from the environment and diet, and if necessary the natural biochemistry is supported with replenishment of essential vitamins, minerals, and microbiota, many patients will improve with a healthy diet, exercise, and rest. Sweating with exercise or sauna may be of benefit, as toxic metals are excreted in sweat [21]. 

Toxic elements unfortunately build up over time in soft tissues and bone, and even when the external source is removed the bioaccumulated toxic elements represent an ongoing endogenous source of exposure, and measures to enhance excretion may be helpful. 

Overall, during chelation therapy mobilization must equal excretion, so adequate hydration and bowel regularity are essential. A variety of products may assist in interrupting enterohepatic recirculation of toxicants, including cholestyramine, charcoal, psyllium, thiolized silica, and others [78]. Pharmaceutical chelating agents may also be considered, to assist with mobilization and excretion.

Chelation therapy, including nonabsorbed agents, should be initiated at a low dose and then gradually titrated to recommended doses according to the individual's response, to avoid the patient's health deteriorating with metal redistribution, other physiological perturbations, or drug intolerance. Mineral status must be monitored during chelation therapy, with panel assays of whole blood or red blood cell essential and toxic minerals, and possibly periodic pre- and postprovocation urinanalyses. Oral or intravenous vitamin and mineral supplementation are important, although mineral supplementation and chelation therapy are antagonistic so are generally not given concomitantly.

DMSA or DMPS are the oral drugs of choice, while EDTA with or without DMPS may be administered intravenously. Concomitant use of NAC or lipoic acid are best reserved until the patient tolerates the full chelator dose, and the metal quantities being excreted have fallen substantially (e.g., to a quarter or fifth of the initial levels).

Allergy-mediated adverse drug reactions have been reported with DMSA and DMPS, and less commonly with CaNa_2_EDTA, so allergy testing may precede chelation therapy. In this context, it is interesting that anecdotally risk of allergy increases with frequency and degree of xenobiotic exposure, which adds further complexities to considerations of type, dose, and frequency of administration of a chelating agent. Clinical experience is that allergies decrease with reduction of the body burden of toxic elements.

## 5. Conclusion

Chelation is the basis of much of the physiology of multivalent cations and of the toxicokinetics and toxicodynamics of heavy metals. Recognizing toxicant contributors to chronic disease and conducting research to evaluate chelation strategies and protocols to assess and address toxic metal bioaccumulation offer potential for inexpensive, safe therapies addressing important root causes of today's most costly, prevalent chronic diseases. Future chelation research should include assessment of both essential and nonessential elements.

## Figures and Tables

**Table 1:** Overview of chelation drugs.

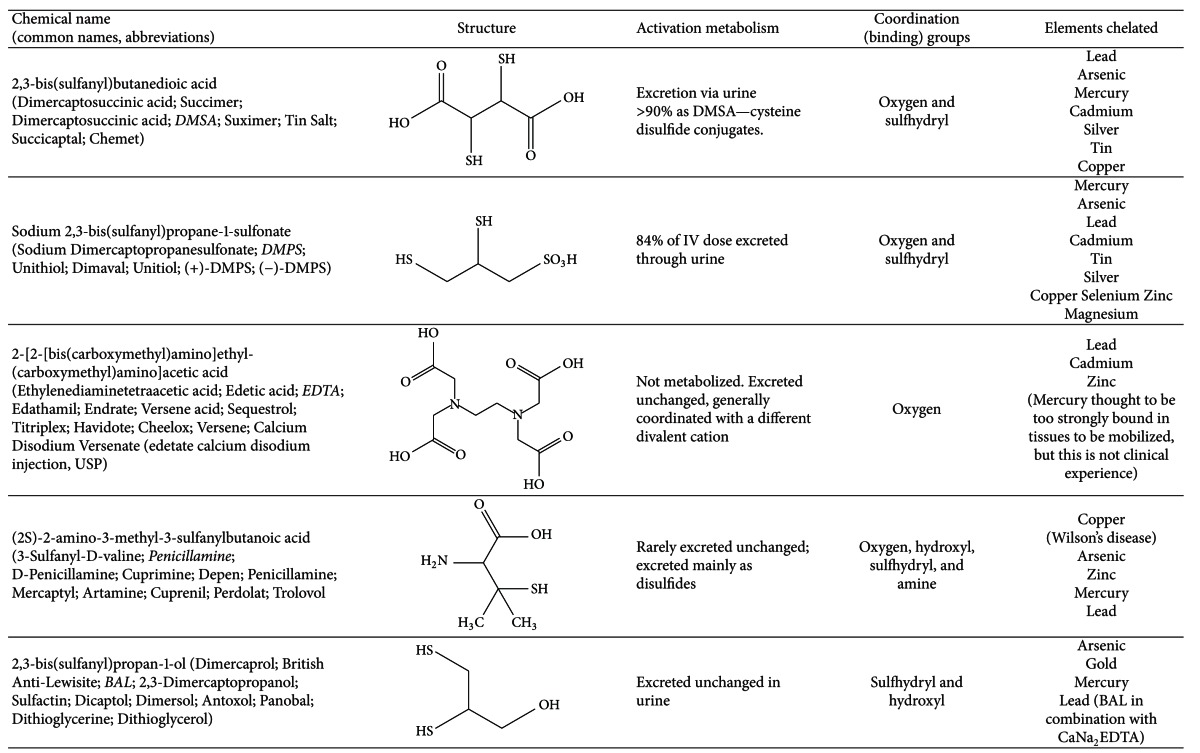

Information from US National Library of Medicine PubChem: http://pubchem.ncbi.nlm.nih.gov/search/search.cgi.
